# Glycoside Mimics from Glycosylamines: Recent Progress

**DOI:** 10.3390/molecules23071612

**Published:** 2018-07-02

**Authors:** Cyril Nicolas, Olivier R. Martin

**Affiliations:** Institute of Organic and Analytical Chemistry, UMR 7311, University of Orleans and CNRS, Rue de Chartres, BP 6759, 45067 Orleans CEDEX 2, France

**Keywords:** addition reactions, glycosylamines, iminosugar-*C*-glycosides, glycomimetics

## Abstract

Glycosylamines are valuable sugar derivatives that have attracted much attention as synthetic intermediates en route to iminosugar-*C*-glycosyl compounds. Iminosugars are among the most important glycomimetics reported to date due to their powerful activities as inhibitors of a wide variety of glycosidases and glycosyltransferases, as well as for their use as pharmacological chaperones. As they provide ready access to these important glycoside mimics, we have reviewed the most significant glycosylamine-based methodologies developed to date, with a special emphasis on the literature reported after 2006. The groups of substrates covered include *N*-alkyl- and *N*-benzyl-glycosylamines, *N*-glycosylhydroxylamines, *N*-(alkoxycarbonyl)-, and *N*-*tert*-butanesulfinyl-glycosylamines.

## 1. Introduction

Carbohydrates are essential ubiquitous molecules that are involved in many fundamental biological events, such as cell-cell recognition or cell adhesion, glycolysis, gluconeogenesis, and signal transduction [1]. They are the most abundant biomolecules on Earth, thus providing very high incentive for the design of glycomimetics as prospective therapeutics [2,3,4,5,6]. Indeed, these analogues may interfere in biochemical pathways wherein carbohydrates play key roles and are associated with pathological disorders [7,8].

*N*-linked glycoconjugates in which the anomeric oxygen of glycosides has been replaced by nitrogen are also natural and valuable sugar-related derivatives [9]. These enclose *N*-glycosyl-amino acids and *N*-glycopeptide derivatives [10,11,12,13,14] (erythropoietin (EPO) is a well-known example), nucleosides, and nucleotides [6,14]. As an aside, *N*-glycoside linkages may also be embedded within many other structurally-diverse natural products such as anthraquinone mycorhodin [15,16], anti-carcinogenic *N*-glycosyl indoles akashines A, B, C [17], staurosporine [18,19,20], and rebeccamycin [21], or ansacarbamitocin antibiotics [22].

Small *N*-glycosyl mimics of glycosides, also known as “glycosylamines”, are per se attractive targets, as they are capable of inhibiting enzymes acting on glycosides [23,24].

**Scheme 1:**
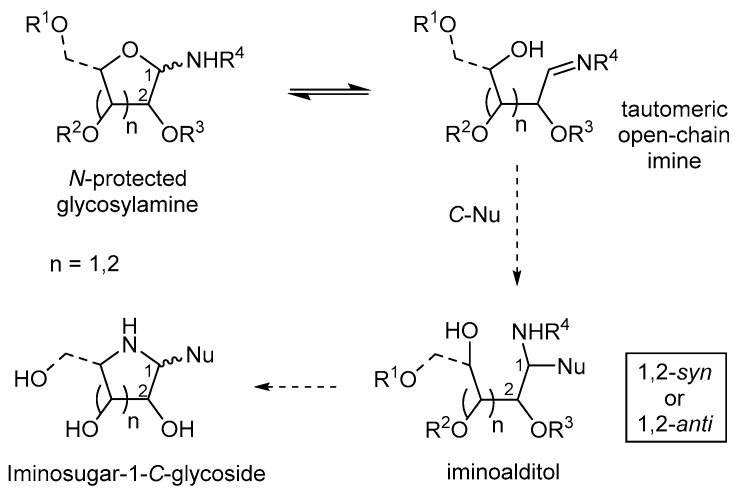
Protected glycosylamines: preparation and use in the synthesis of iminosugar-*C*-glycosides.

Interestingly, like sugars, some of these *N*-glycosyl compounds exhibit mutarotation [25]. They rearrange to a tautomeric open-chain imine and are, therefore, capable of reacting with a variety of carbon nucleophiles to provide 1,2-*syn* or 1,2-*anti* aminoalditols in good yields and good levels of stereoselectivity. After activation of the pendant alcohol, cyclization, and further deprotections, related iminosugar-*C*-glycosyl compounds are obtained in good yields (Scheme 1) [26].

Iminosugars are found in a wide variety of microorganisms and plants. These form, probably, the most important class of carbohydrate mimics reported to date [27,28,29,30]. However, one of the major drawbacks associated with imino analogues of glycosides is their instability caused by the lability of the *N*,*O*-acetal function, which prevents their use as biological probes or drug candidates. As glycoside mimics, 1-deoxyiminosugars have, thus, gained considerable importance as bioactive molecules. A typical example is 1-deoxygalactonojirimycin (Galafold^®^), now used to treat Fabry disease [31]. However, unlike this notable example, absence of structural and configurational information of normal (α- or β-linked) glycosides may prevent their use as drugs. This problematic feature could, to some extent, be prevented by relocating pieces of structural information in the nitrogen substituent. For instance, it may be the case of *N*-[2-hydroxyethyl]-1-deoxynojirimycin (Diastobol^®^) [32], and *N*-butyl-1-deoxynojirimycin (Zavesca^®^) [33] that are used for the treatment of diabetes mellitus type 2 and Gaucher disease, respectively. By analogy, iminosugar-*C*-glycosides may, thus, be more significant as stable glycoconjugate or oligosaccharide mimetics of biological and therapeutic interest, since a strong and non-hydrolyzable C–C bond has replaced the labile glycosidic linkage of real iminoglycosides [26,27].

Iminosugar-1-*C*-glycosides are powerful inhibitors of a wide variety of glycosidases [27,28,29,30], and glycosyltransferases down to a femtomolar range [34,35,36]. They may also be employed as pharmacological chaperones to treat deficiencies characterized by improperly folded proteins [37].

Their chemical syntheses and therapeutic applications are well documented [26]. Although recent progress in their de novo preparation through the diversity oriented synthetic approach by elegant asymmetric organocatalyzed processes [38,39,40] have been reported, one of the best methods remains the addition of *C*-Nu to *N*-glycosylamines.

In this review, we have compiled the most significant glycosylamine-based -methodologies developed to date, with emphasis on the literature reported after 2006. They involve *N*-alkyl- and *N*-benzyl-glycosylamines, *N*-glycosylhydroxylamines, *N*-(alkoxycarbonyl)-, and *N*-*tert*-butanesulfinyl-glycosylamines.

## 2. *N*-(Benzyl)- and Other *N*-(Alkyl)-*N*-Glycosides

Some aspects of the chemistry of *N*-benzylglycosylamines have been reviewed by Behr and Plantier-Royon in 2006 [41], and recent progress in this area will be outlined here.

**Scheme 2:**
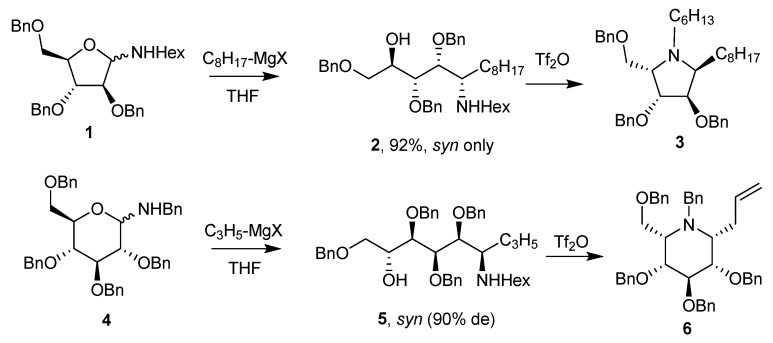
Addition of Grignard reagents to *N*-benzylglycosylamines.

Pioneering studies on glycosylamines have been reported by Nicotra and coworkers since 1989 [42]. These authors have shown, for the first time, that the addition of Grignard reagents to *N*-benzyl and *N*-alkyl glycosylamines derived from perbenzylated pentofuranoses or hexopyranoses followed by a simple cyclization procedure afforded a short and convenient approach to imino-*C*-glycosides in the pyrrolidine and piperidine series [43,44]. The procedure is illustrated in Scheme 2 (see compounds **1**–**6**) from a d-arabinofuranosylamine, using octylmagnesium bromide, and from a d-glucopyranosylamine using allylmagnesium bromide, and cyclization promoted by reacting the intermediate amino alditol with triflic anhydride.

In the *gluco* series, the final product is a mimic of an α-glycoside (as in **6**), whereas in the *manno* series, the cyclized product is a mimic of a β-glycoside [43,44]. Also it is important to note that cyclization at a secondary position, as in **2** and **5**, leads to an *inversion* at this position, d-arabino*f* substrates leading to l-xylo*f* products (as in **3**) and d-gluco*p* substrates leading to l-ido*p* products (as in **6**).

The Nicotra group further investigated this process to prepare the significant DNJ derivatives (d-*gluco* epimer of **6**) [45,46]. This requires the oxidation at C-5 of the addition intermediate (as in **5**) and cyclization by a reductive amination, a reaction known to favor axial hydride delivery and, hence, formation of the “d” stereoisomer from **5** [47]. This sequence was made possible providing the nitrogen atom was protected by a Fmoc group during oxidation.

The Nicotra procedure was also used more recently by other groups. 1-*C*-allyl iminosugar derivatives in the α-d-*gluco*, β-l-*ido*, and α-d-*xylo* series were prepared by Overkleeft et al. [48]. While the synthetic sequences are similar to those already described, the Leiden group chose to use *N*-*p*-methoxybenzyl glycosylamines as substrates in order to facilitate the selective cleavage of the *N*-alkyl substituent and replace it by a carbamate for further functionalization of the allyl group mainly by cross-metathesis. Allylation of the *N*-*p*-methoxybenzyl glucosylamine was also exploited by Vankar et al. [49] in order to reach an advanced synthetic intermediate in their synthesis of novel hydroxylated indolizidines and pyrrolizidines. The addition of vinyl-magnesium bromide to *N*-benzyl pentopyranosylamines (d-*xylo*, l-*arabino*) was a key step in recent work of the Fleet’s group leading to the total synthesis of calystegines B_2_ and B_3_ [50], as well as of the Peczuh work aiming at amino septanosyl conjugates [51,52]. An improvement of the formation of glycosylamines (i.e., faster reaction times and better yields) using iodine in the presence of imidazole was reported by Chagnault et al. [53].

**Scheme 3:**
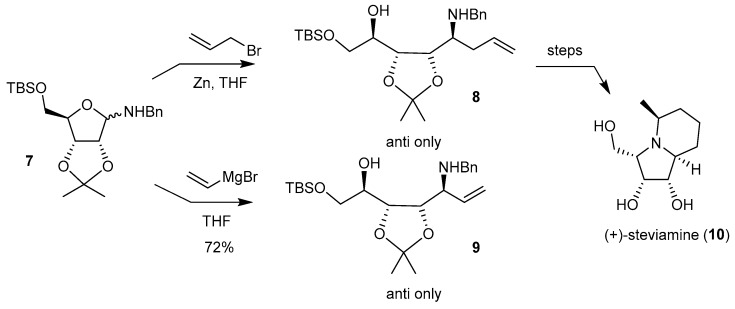
Addition of Grignard reagents to *N*-benzylglycosylamines in the ribofuranose series.

From a stereochemical viewpoint, the addition of the organometallic reagent appears to be controlled by the group at C-2 (usually an *O*-benzyl group) of the substrate (Cram chelate) leading to the 1,2-*syn* diastereoisomer, predominantly or exclusively and, hence to a 1,2-*cis* configuration after cyclization to an iminosugar. On the other hand, the *anti-*configuration was only observed in glycosylamines derived from 2,3-*O*-isopropylidene ribofuranose derivatives and related scaffolds, which afforded iminosugars with a 1,2-*trans* configuration (Rao et al. [54,55,56,57] (see for example Scheme 3, compounds **7**−**10**), Behr et al. [58,59,60]). For a rationale, see [54].

**Scheme 4:**
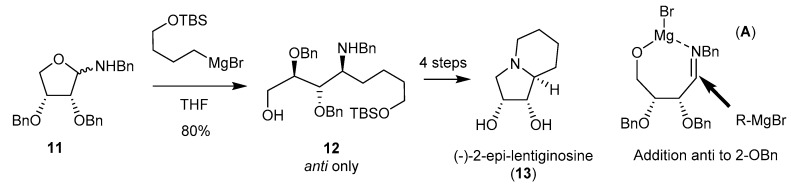
Addition of Grignard reagents to *N*-benzylglycosylamines in the erythrofuranose series.

As an exception, the *anti* addition product was observed by Zhuang et al. [61] in the reaction of the glycosylamine derived from 2,3-*O*-isopropylidene-d-erythrofuranose with a Grignard reagent (Scheme 4). It was suggested that the reaction takes place by way of a seven-membered-ring complex (e.g., **A**).

**Scheme 5:**
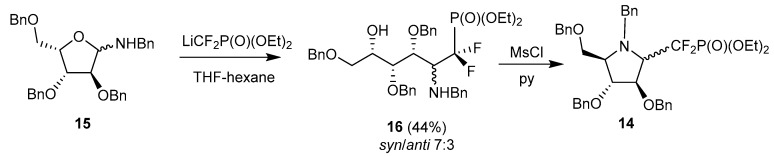
Addition of lithium difluoromethylphosphonate to an *N*-benzyl furanosylamine.

Several research groups have adopted this strategy to reach biologically-significant iminosugar derivatives. In early studies, Behr, Guillerm and coworkers took advantage of this methodology to prepare potential glycosyl transferase inhibitors; in particular they investigated the addition of lithium difluoromethyldiethylphosphonate to *N*-benzylpentofuranosylamines, as an approach to glycosylphosphate analogs, such as **14**, from L-xylofuranosylamine **15** (Scheme 5) [62].

**Scheme 6:**
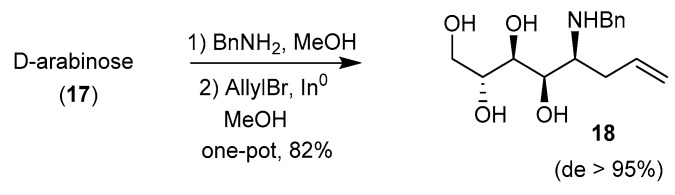
Indium-mediated allylation of a free pentose.

These phosphonates were later deprotected and tested as antifungal agents, together with other pyrrolidines related to DMDP and obtained by the Nicotra’s procedure [63]. The same group also took advantage of the addition of allyl Grignard reagent to glycosylamine **15** and to a related 5-deoxy-l-lyxofuranosylamine to prepare 6-deoxy-homoDMDP and iminosugar-ferrocene conjugates, respectively [59,64]. Interestingly, Behr et al. demonstrated in 2012 that the allylation of free glycosylamines could be achieved using indium metal in MeOH, with excellent *syn* stereoselectivity, and the two steps could be achieved in one pot (Scheme 6) [65]. Application of this protocol to glycosylamines derived from (*R*)- and (*S*)-α-methylbenzylamine revealed that the chiral group did not mediate the stereoselectivity of the reaction.

**Scheme 7:**
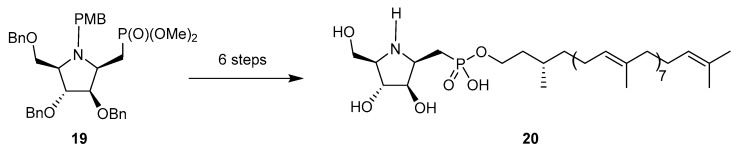
Synthesis of the DPA mimic.

The methylenephosphonate analogs of **14** (in both configurations) were prepared by Eustache et al. starting from the *N*-*p*-methoxybenzyl glycosylamine equivalent of **15** [66]. Interestingly, the addition of the lithium methylphosphonate was greatly facilitated when the glycosylamine was first treated with BF_3_·Et_2_O. The ‘β’anomer **19** was then converted in six steps into compound **20** (Scheme 7), a remarkable mimic of DPA (β-d-arabinofuranosyl-1-monophosphoryl decaprenol), the glycosyl donor involved in the biosynthesis of arabinans in mycobacteria. This compound is endowed with good MIC values toward mycobacteria, comparable to ethambutol.

**Scheme 8:**
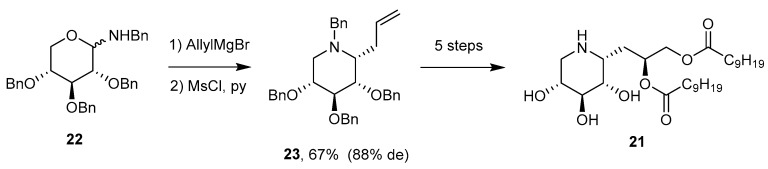
Synthesis of a Glucosylceramide Mimic.

In our own work, we have prepared a series of 1-*C*-alkylated imino-l-iditols using Nicotra’s procedure, in order to compare the activity of these compounds as β-glucocerebrosidase inhibitors with the α-d-gluco epimers [67]. Furthermore, the synthesis of new glucosylceramide mimics based on an iminoxylitol core (e.g., **21**, Scheme 8) was achieved form *N*-benzyl-d-xylopyranosylamine **22** by the stereoselective addition of allylMgBr, cyclization (to give **23**) and elaboration of the allyl group into a 2,3-di-*O*-acyl or 2,3-di-*O*-alkylglyceryl residue [68]. Compound **21**, a potent inhibitor of this enzyme (*K*_i_ = 1.8 nM), was found to exhibit a significant activity as chaperone of the mutant form of β-glucocerebrosidase carrying the L444P mutation. This mutation is responsible for the neuronopathic form of Gaucher disease, for which there is currently no treatment.

Compounds such as **21** which act as pharmacological chaperones, constitute new leads for the treatment of this severe form of Gaucher disease, which cannot be treated by Enzyme Replacement Therapy. In more recent work, with the goal of preparing iminosugar derivatives carrying a 1-*C*-propargyl group for further functionalization, we have investigated the addition of TMS-propargyl bromide to the *N*-benzyl-d-xylofuranosylamine (ent-**15**); best conditions consisted in using Zn dust and performing the reaction under ultrasound activation [69]. The reaction gave the expected product (*syn* relative configuration, 60% d.e.) in 62% yield after cyclization, but the conditions were found to be difficult to reproduce, and better results were obtained from the corresponding *N*-sulfinyl glycosylamines (vide infra).

**Scheme 9:**
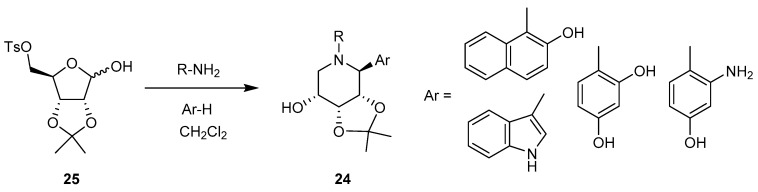
One-pot process leading to *C*-arylated iminosugars.

A number of interesting methodologies involving in situ formation of the glycosylamines have been reported. In particular, Baskaran and coworkers have developed elegant methodologies in which the glycosylamine is trapped by various nucleophiles:

Electron-rich aromatic groups [70]: a great diversity of C-aryl iminosugars **24** have been generated from **25** by the general methodology outlined in Scheme 9.

**Scheme 10:**
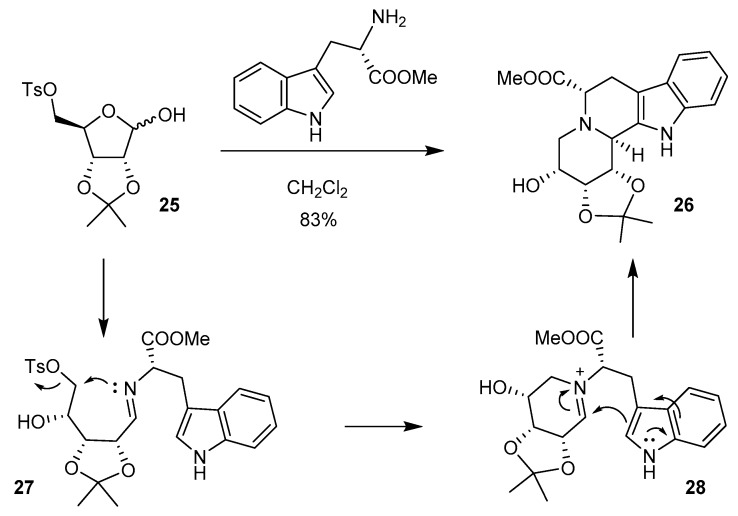
Synthesis of a tetracyclic iminosugar derivative using tryptophane methyl ester.

In addition, using an amine carrying an electon-rich aromatic substituent, the procedure led, in one step, to innovative polycyclic systems, such as **26** (Scheme 10).

In these reactions, the in situ-generated imine **27** undergoes intramolecular *N*-alkylation by the tosylate leading to a cyclic iminium cation **28**, which is sufficiently reactive to promote an electrophilic substitution of the electron-rich aromatic compounds. All reactions occur in high yield (68–92%), and high stereoselectivity, the 1,2-*trans* stereoisomer being exclusively formed.

**Scheme 11:**
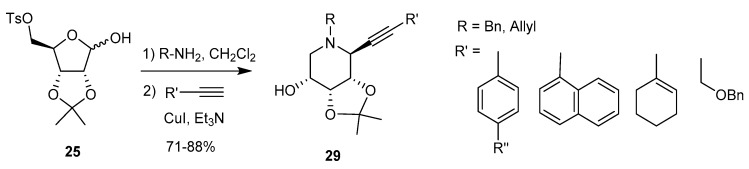
Synthesis of 1-C-alkynylated iminosugars.

Alkynyl anions [71]: A further extension of this work allowed the introduction of an alkynyl group at the ‘pseudoanomeric’ position: in situ formation of the cyclic iminium ion as before followed by reaction with a terminal alkyne in the presence of a Cu(I) salt gives access to 1-*C*-alkynyl piperidine iminosugar derivatives **29**, in high yield and complete stereoselectivity (Scheme 11).

Various polycyclic systems were obtained from reactions between the nitrogen substituent (allyl, *o*-bromobenzyl) and the alkynyl group. The authors also showed that pyrrolidine derivatives could be obtained by a similar procedure starting from 4-*O*-mesyl-2,3-*O*-isopropylidene-l-rhamnopyranose, cyclization occurring at C-4 of this hexopyranose.

**Scheme 12:**
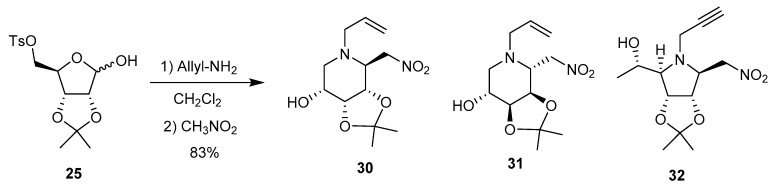
Synthesis of 1-*C*-nitromethyl iminosugars.

Nitromethyl anions [72]: In a very simple, two-step one-pot procedure, piperidine iminosugars carrying a 1-*C*-nitromethyl group were obtained from d-ribo*f* tosylate **25** by reaction with a primary amine in the presence of Et_3_N, followed by the addition of the nitromethyl anion to the in situ*-*generated iminium cation. This led to nitromethyl derivatives, such as **30** (Scheme 12), with 1,2-*trans* diastereoselectivity exclusively. By similar reactions from the d-*lyxo* isomer of **25**, the epimers **31** were obtained, still with dominance of the 1,2-*trans* isomer (d.r. = 3.1)

Pyrrolidine analogs (e.g., **32**) were prepared from 4-*O*-mesyl-2,3-*O*-isopropylidene-l-rhamno-pyranose by the same sequence of reactions. Owing to the rich chemistry of the nitromethyl group, 1-*C*-nitromethyl iminosugar derivatives constitute precursors of a wide variety of further glycoside mimics, as well as to novel polycyclic compounds. Several examples of further functionalization/cyclization by reactions of the nitromethyl group with the nitrogen substituent (allyl, propargyl) were reported by Baskaran and coworkers [71].

**Scheme 13:**
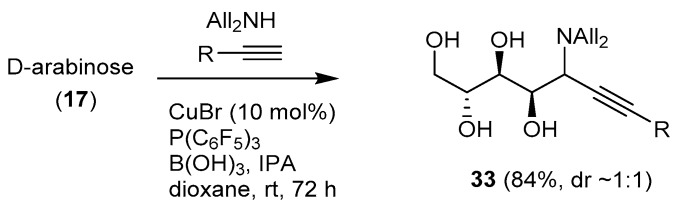
Cu-mediated alkynylation of an in situ-generated furanosylamine.

A Cu(I)-catalyzed aminoalkynylation of unprotected aldoses was reported by Kanai et al. [73] (Scheme 13). The one-pot reaction of free sugars (pentoses, d-galactose, l-fucose) with diallylamine, a terminal alkyne, catalytic CuBr, a boron reagent (boric acid), and a ligand (P(C_6_F_5_)_3_) afforded the corresponding chain-extended aminoalditols **33** in good to very good yields and with rather low diastereoselectivity (with some exceptions, the *anti* product being predominant).

The reaction was applied to substrates of biological significance, including a biotinylated alkyne derivative. The addition products however were not cyclized owing to the unprotected nature of the substrate.

**Scheme 14:**
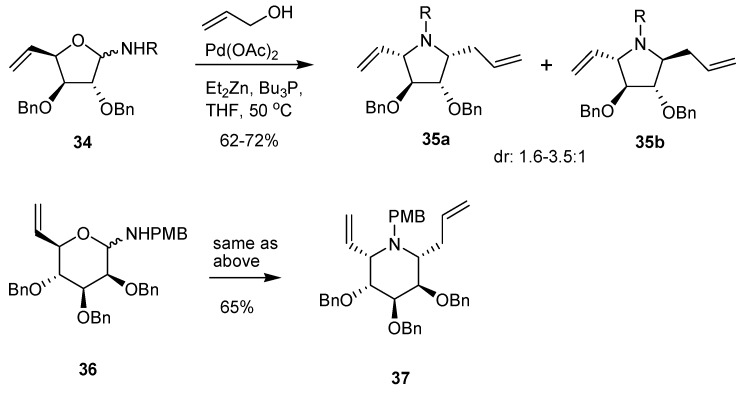
1-*C*-allylation of 4- or 5-*C*-vinylated glycosylamines.

In very recent work, Rao et al. reported an interesting Pd-mediated double allylation process in which the C-N bond is created in the same step as the C-C bond [74]. This procedure requires a substrate (furanosyl- or pyranosylamine) carrying a vinyl group at C-4 or C-5, respectively. Thus, for example, glycosylamine **34** was submitted to reaction with allyl alcohol in the presence of diethylzinc, tributylphosphine, and catalytic Pd(II) acetate, in THF at 50 °C for 24 h, to give directly the cyclized 1-*C*-allyl, 4-*C*-vinyl pyrrolidine **35**, with low stereoselectivity, however (Scheme 14).

The reaction has also been applied successfully to pyranosylamines. For example, the 6,7-unsaturated mannopyranosylamine **36** gave the 1-*C*-allylated L-*gulo* iminoalditol **37** as a single stereoisomer (Scheme 14). The final products obtained (e.g., **37**) contain two vinyl groups which were combined by ring-closing metathesis to form several analogs of the calystegins. The mechanism involves a Pd-mediated formation of a nucleophilic allylzinc species which react with the open-chain form of the glycosylamine to give an intermediate containing an allylic alcohol function presumed to be in the form of a ZnEt salt. This undergoes an intramolecular Tsuji-Trost electrophilic allylation of the amine function, thus leading to the formation of the C-N bond and ring-closure.

**Scheme 15:**
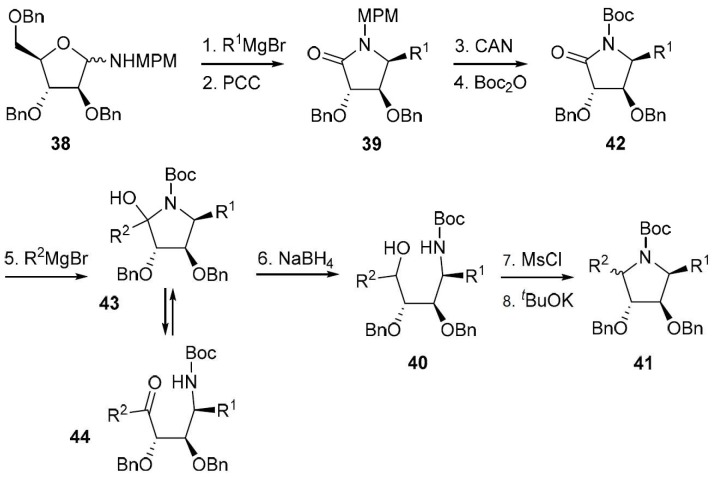
Synthesis of 2,5-dialkylated pyrrolidine 2,3-diol.

Finally, a modified version of the Nicotra’s procedure was reported in the 1990s by Yoda et al. [75,76]. In this work, the product resulting from the addition of a Grignard reagent (R^1^ = C_4_H_9_, C_9_H_19_, Bn) to a glycosylamine such as **38** was submitted to an oxidative chain shortening leading to a carboxylic acid derivative which underwent spontaneous cyclization to a lactam (e.g., **39**) (Scheme 15). The interest of this procedure is the possibility to use the lactam for a second alkylation step, by way of the addition of an organometallic reagent (e.g., R^2^ = C_4_H_9_, C_9_H_19_, Bn), reduction of the resulting hemiaminal to give **40**, and cyclization by an S_N_2 process.

This sequence leads to pyrrolidine derivatives carrying two different R groups at C-1 and C-4, such as **41**. The authors used this procedure to prepare the natural product (+)-*preussin* [76].

## 3. *N*-Benzyl-*N*-Glycosylhydroxylamines

### 3.1. Synthesis

In general, 1,2-*anti* aminoalditol derivatives could be synthesized in a more diverse and effective manner via the addition of lithium and magnesium reagents to *N*-benzyl-*N*-glycosyl-hydroxylamines. This approach is indeed more general than the ones described for *N*-(benzyl)-*N*-glycosides which require the use of 2,3-*O*-isopropylidene-protected *ribo* or *lyxo* glycosylamines or the isolation of the minor diastereomer of the open-chain adducts.

*N*-benzyl-*N*-glycosylhydroxylamines were frequently prepared by heating mixtures of *N*-benzylhydroxylamine and sugar hemiacetals at 110 °C for 30–60 min under-solvent free conditions [77,78,79]. Alternatively, they were prepared by stirring a suspension of *N*-benzylhydroxylamine hydrochloride, 3 Å molecular sieves and the hemiacetal derivatives in dry pyridine at room temperature [80].

Various *N*-benzyl-*N*-hydroxy-glycosylamines **45a**–**45h** (see Table 1), derived from furanoses and pyranoses, have been prepared and isolated (up to multigram scale) in moderate to good yields (61–88%).

### 3.2. Addition Reactions

**Scheme 16:**
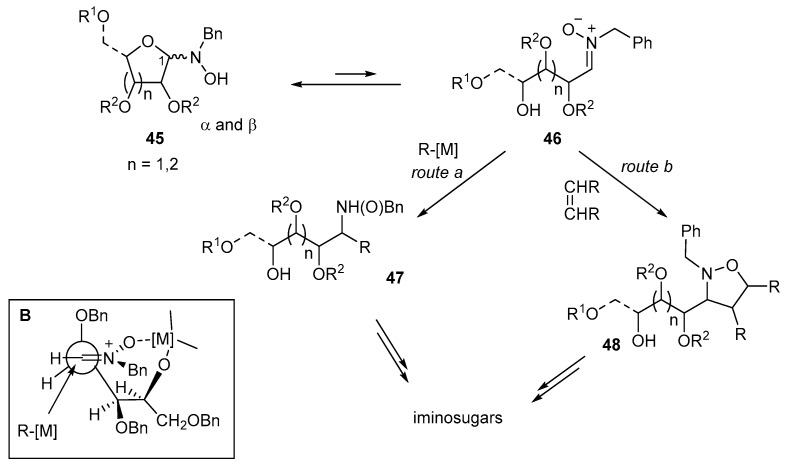
Synthetic approaches to iminosugars using glycosylhydroxylamines.

The two anomers of these sugar-hydroxylamines exist in equilibrium with the masked open-chain nitrones (**46**). Although the equilibrium is largely or completely shifted to cyclic hydroxylamines, these forms undergo reactions with various lithium and magnesium reagents at low temperature to give the corresponding adducts (**47**), albeit often in inseparable mixtures of diastereomers (*route a*, Scheme 16) [77,78,80,81].

The results of the addition reactions are summarized in Table 1. In general, an excess of organometallic species in Et_2_O or THF was added to cooled solution (−75 or −30 °C) of **45a**–**45h** in THF to provide **47a**–**47h** in good yields (52–95%) and moderate to high levels of diastereoselectivity in favor of the 1,2-*trans* adducts (see entries 1–9, 11, 14) [77,78,81].

In contrast, hexopyranosylamines **45f**, **45g**, and **45h** did not react with 2-lithiothiazole under these conditions [79]. Treatment of these hydroxylamines with 5 equiv. of 2-thiazolylmagnesium bromide in THF at 0 °C was mandatory to afford the diastereomers of thiazolylalkyl-hydroxylamines **47f**, **47g**, and **47h** in good overall yields, but modest selectivities (see entries 10, 13 and 16). Of note, the major diastereomers of **47f** and **47h** were both *syn*-adducts, whereas the main product of **47g** was an *anti*-adduct [79]. Obviously, the carbon stereocenter adjacent to the nitrone group affects the selectivity of these addition reactions. Moreover, a 1:1 mixture of diastereomeric adducts were obtained with allylmagnesium bromide (entries 12 and 15). The stereoselectivity was not improved by lowering the reaction temperature to −50 °C, while, at this temperature, the yields dramatically decreased [78].

As a rule, the *anti* selectivities may be rationalized by a preferential conformation (**B**) adopted by the open-chain nitrone form **46** due to the metal coordination, involving the nitrone oxygen and the free hydroxyl group. As a consequence, the addition occurs to the less hindered side of this complex to give the *anti*-product (Scheme 16).

### 3.3. Cyclizations

Due to the usual difficulty in separating the two diastereomers of compounds **47**, the open-chain products were often subjected to numerous synthetic sequences. In general, reductive *N*-dehydroxylation using a Zn–Cu couple [82] was achieved in good yield (ca. 90%). Then, the resulting benzylamino-1-deoxyalditol derivatives **47** were transformed into pyrrolidine and piperidine iminosugars following standard activation of the free hydroxyl group and cyclization. MsCl in the presence of Et_3_N was used for the synthesis of *N*-benzyl-*N*-glycosides. For the cyclization of compounds **47f**–**h** which do not cyclize under the present conditions, a catalytic amount of tetramethylethylenediamine (TMEDA) was added as a promoter [83], followed by heating the crude product in MeCN at 85 °C.

Overall, collections of 1,2-*trans* iminosugar-*C*-glycosides were successfully prepared. Through the stereoselective addition of 2-lithiothiazole and 2-thiazolylmagnesium bromide, access to dideoxyiminoheptitols (e.g., piperidine homoiminosugars) from pyranoses was conveniently achieved via a formal one-carbon chain elongation [79]. This thiazole-to-formyl unmasking protocol was further utilized to generate aza-*C*-disaccharides as methylene isosteres of *O*-disaccharides [77].

**Figure 1:**
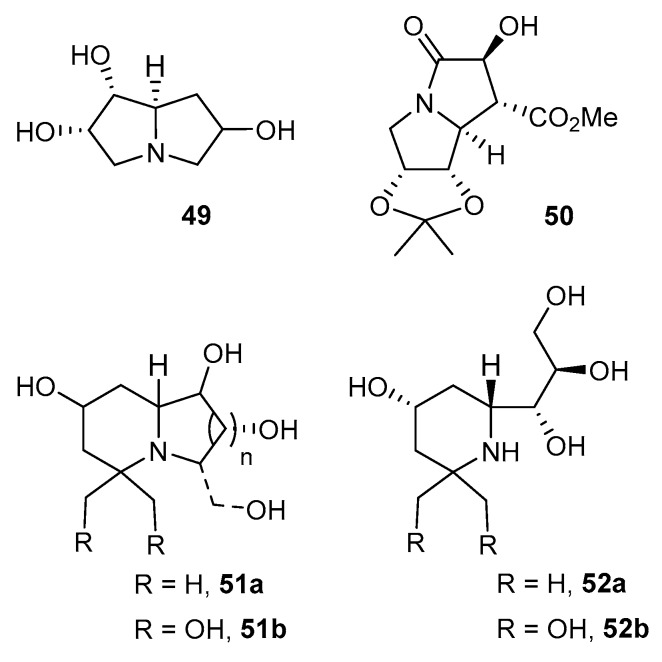
Iminosugar-*C*-glycosyl compounds from *N*-glycosylhydroxylamines.

As outlined in Scheme 16 (*route b*), *N*-glycosylhydroxylamines may also react as masked nitrones in 1,3-dipolar cycloaddition reactions (see **48**). This synthetic approach was undertaken by the group of Argyropoulos to synthesize enantiomerically-pure trihydroxypyrrolizidines of type **49** [84], and by Goti and co-workers to prepare highly-functionalized pyrrolizidine **50** [85]. The nitrones could serve further to react via [1,4]-sigmatropic rearrangement to construct iminosugars of various heterocyclic cores (e.g., **51a**, **51b**, **52a**, **52b**, Figure 1) [86].

## 4. *N*-(Alkoxycarbonyl)-*N*-Glycosides

1,2-*Syn* aminoalditols may also be efficiently synthesized through the addition of silylated nucleophiles to *N*-(benzyloxycarbonyl)-glycosylamines under Lewis acid catalysis, opening an approach to iminosugar-*C*-glycosides carrying a greater diversity of aglycon moieties (e.g., allenyl, oxoalkyl, etc.).

### 4.1. Synthesis

Studies on the addition of silicon-based nucleophiles to semicyclic *N*,*O*-acetals possessing an exocyclic nitrogen atom protected by an alkoxycarbonyl group were pioneered by Kobayashi on simple substrates [87,88], and subsequently applied to the sugar series [89].

**Scheme 17:**
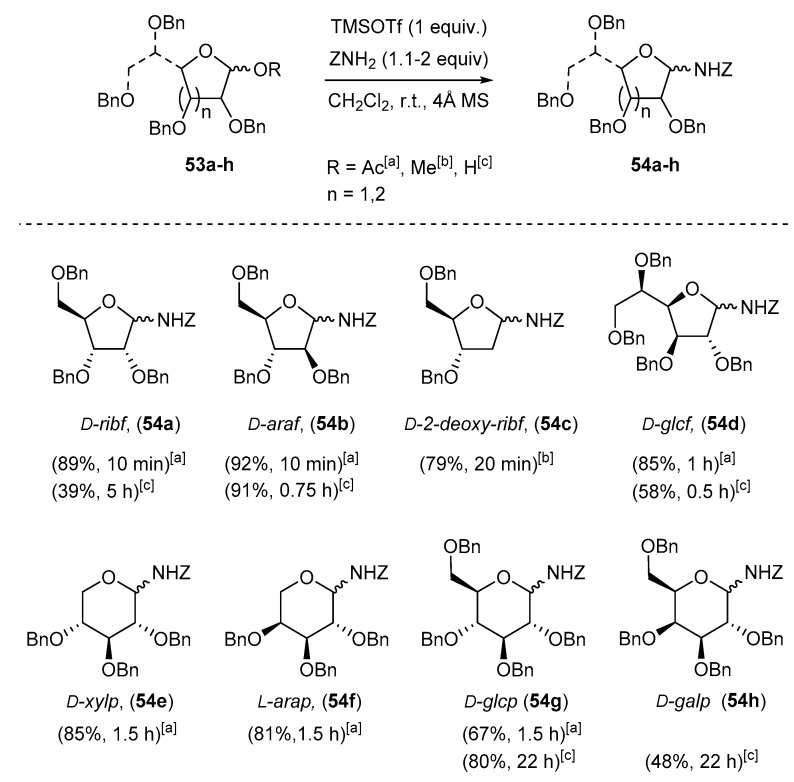
Synthesis of *N*-(benzyloxycarbonyl)-*N*-glycosides **54a**–**h** [89,93].

Carbohydrates derived *N*-benzyloxycarbonyl-*N*-glycosides are typically protected by *O*-benzyl substituents (Scheme 17). Their preparation proceeds through dropwise addition of trimethylsilyl trifluoromethanesulfonate (TMSOTf) to a suspension of benzyl carbamate (1.1–2 equiv.) and related glycosyl acetates **53** in CH_2_Cl_2_ at room temperature [89,90,91,92]. Since benzyl carbamate is a weak nucleophile and glycosylamines are unstable under aqueous conditions, addition of 4 Å molecular-sieves (MS) is essential to prevent the formation of hydrolyzed products [89].

A mixture of α- and β-*O*-benzyl-*N*-benzyloxycarbonyl glycosylamines (d-ribo*f*, d-ara*f*, d-glc*f*, d-glc*p*, d-xyl*p*, l-ara*p*, etc.) were isolated in good yields (>67%). 2-Deoxy-d-glycofuranosyl-amines were obtained following the same conditions from the *O*-methyl glycoside derivatives (e.g., **54c**) [89]. The choice of the protective groups may be further extended to acetates, but it was shown afterwards that a 2-*O*-acetyl group is detrimental to the chain extension reaction. An electron withdrawing group at the α-position will, of course, retard the formation of the assumed *N*-acyliminium intermediate [89].

**Scheme 18:**
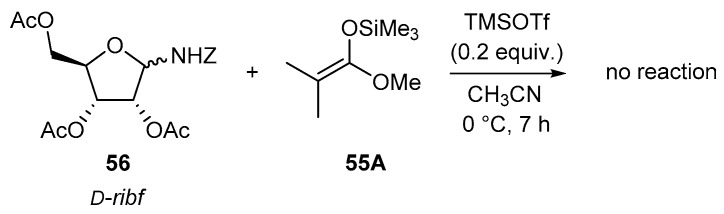
Attempted ring-opening reactions of *O*-acetyl-protected furanosylamines.

**Scheme 19:**
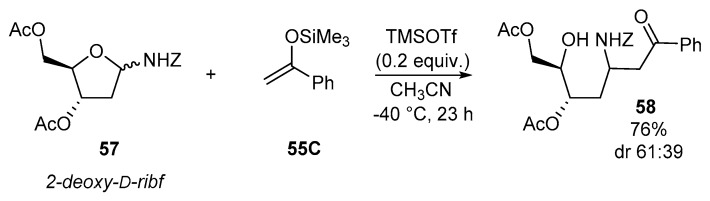
Ring-opening reactions of *O*-acetyl-protected 2-deoxy furanosylamines.

Indeed, no reaction was observed by the addition of silyl enolate **55A** to benzyl-(2,3,5-tri-*O*-acetyl-d-ribofuranosyl)carbamate **56** (Scheme 18), whereas the analogous deoxyribose derivative **57** underwent the addition reaction in good yield (76%) albeit with low stereoselectivity (dr 61:39) [89] (Scheme 19). *O*-silylated groups could also possibly be employed, but no example has been reported in the sugar series [88].

Using these conditions, hexopyranose-based *N*-(benzyloxycarbonyl) *N*,*O*-acetals were also obtained, but in yields lower than for the more entropically-favored five-membered ring sugars and pentopyranose derivatives. *O*-Benzyl-*N*-(2,3,4,6-tetra-*O*-benzyl-d-glucopyranosyl)carbamate **54g** was, thus, obtained in moderate yield (67%) from 1-*O*-acetyl-2,3,4,6-tetra-*O*-benzyl-d-glucopyranose **53g** (R = H, Scheme 17) [89].

Alternatively, conversion into *O*-acetyl glycosides is not mandatory. The amination protocol could be performed directly from free sugar hemiacetals using similar conditions [93], although the expected glycosylamines were obtained after longer reaction time (0.75–22 h) (see Scheme 17, above).

It is worth noting that carbonylated glycosylamines have low stability under acidic conditions, limiting their isolation by SiO_2_-column chromatography, mainly for hexopyranosides. They may also act as activated glycosyl donors, leading to *C*-glycosyl compounds. As a rule, the stability order is furanosyl > pentopyranosyl > hexopyranosyl derivatives.

### 4.2. Addition Reactions

**Figure 2:**
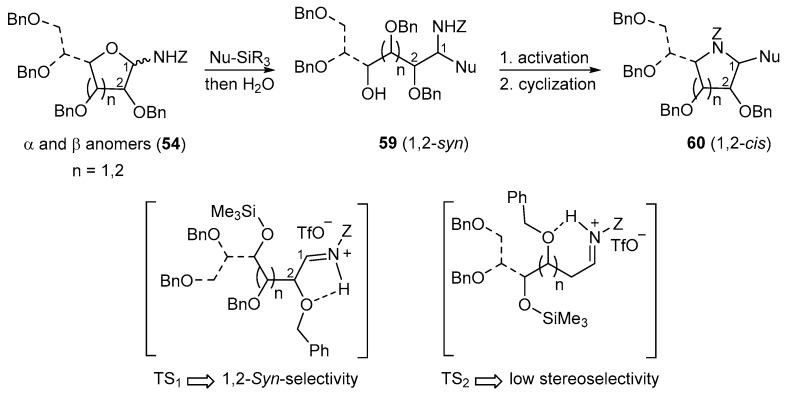
Synthesis of iminosugar-*C*-glycosides from *N*-Z-glycosylamines and proposed transition states for the acyclic iminium ion intermediate.

*N*-Benzyloxycarbonyl-*N*-glycosides (**54**) undergo Lewis acid-catalyzed ring-opening reactions with silylated nucleophiles (Nu-SiR_3_) to give related 1,2-*syn*-aminoalditols (**59**) with good to high diastereoselectivity through Cram chelate transition states (TS_1_, Figure 2). As for *N*-alkyl- and *N*-benzyl-*N*-glycosides, they then provide 1,2-*cis*-iminosugar-*C*-glycosyl compounds (e.g., **60**) in good yields after activation and a cyclization reaction (Figure 2).

**Scheme 20:**
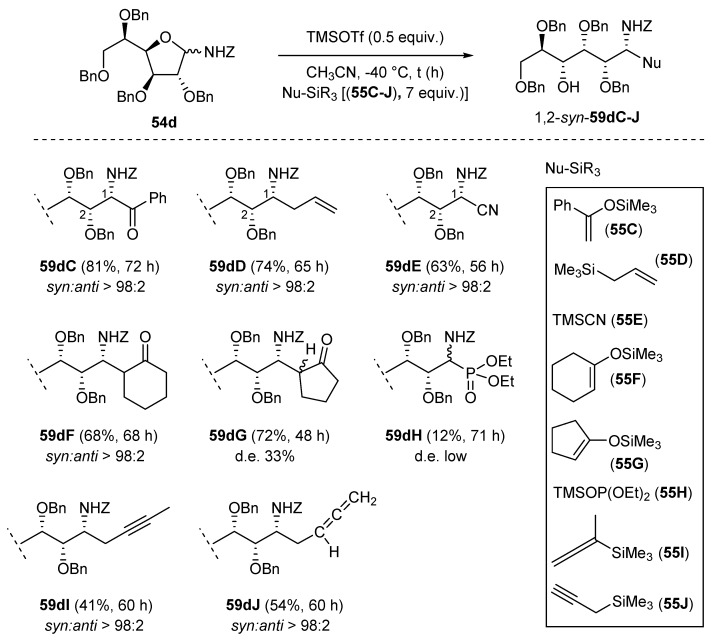
Addition of silicon reagents **55C**–**J** (Nu-SiR_3_) to *N*-glucofuranosylamine **54d** [90,91].

Addition reactions performed in the furanose series were carried out with a sub-stoichiometric amount of TMSOTf and an excess of the silylated nucleophile in CH_3_CN at low temperature (−40 °C). The main examples are shown below (Table 2, and Scheme 20) [89,90,91]. Importantly, no reaction of **54a** and allyltrimethylsilane **55D** was observed, while 2-deoxy-d-ribo derivative **54c** showed no diastereoselectivity when reacted with acetophenone-derived silyl enol ether **55C**. This further highlights the rationale of assuming Cram chelated transition states during the nucleophilic addition process involving the *O*-alkyl substituent at C-2 (see Figure 2, TS_1_ vs. TS_2_) [89].

The synthetic value of this reaction was extended to several functionalities as various important motifs were introduced on an *N*-glucofuranosylamine derivative **54d** (e.g., TMSCN (**55E**), the silyl enol ether of cyclohexanone (**55F**), and cyclopentanone (**55G**), etc., see Scheme 20).

It is noteworthy that a single stereoisomer was formed at the alkylation site α to the carbonyl group in the cycohexanone derivative **59dF**, whereas a mixture of two stereoisomers (ratio 2:1) was isolated for the cyclopentanone analogue **59dG**. A lower yield (12%) and a low diastereoselectivity were observed for the addition of diethyl trimethylsilyl phosphite, although it was possible to isolate the related α-amino phosphonate product **59dH**. Unsaturated aliphatic chains could also be introduced as alkyne or allene moieties in moderate yields using 3-trimethylsilyl-1,2-butadiene **55I** or propargyltrimethylsilane **55J** as reagents. These compounds are of particular interest as synthetic precursors of disaccharide mimics [90,91].

**Scheme 21:**
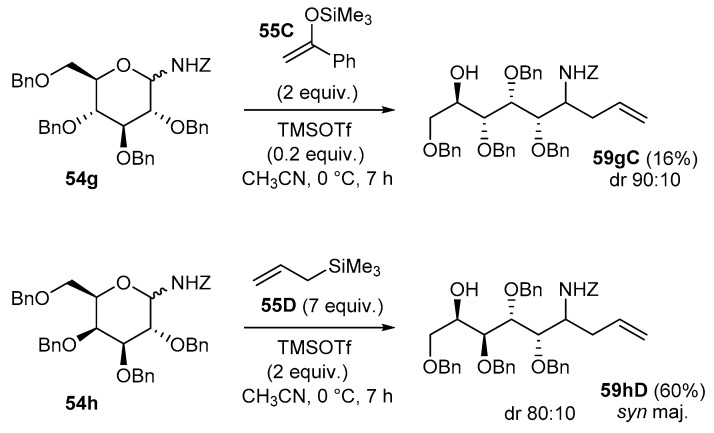
Ring-opening reactions of *N*-Z-hexopyranosylamines **54g** and **54h** [89,94].

In the pyranose series, however, under the typical reaction conditions, the limited stability of the six-membered glycosylamines commonly preclude the addition reaction even after prolonged reaction time. As depicted in Scheme 21, this was observed for the addition of the silyl enol ether of acetophenone (**55C**) to the *N*-Z-protected d-glucopyranosylamine **54g** which gave the addition product **59gC** in 16% yield [89]. However, the addition of allylTMS **55D** onto d-galactosamine **54h** could be achieved using 2 equiv. of TMSOTf in a yield of about 60% (**59hD**) when the isolated reaction mixture was submitted a second time to the same conditions [94].

**Scheme 22:**
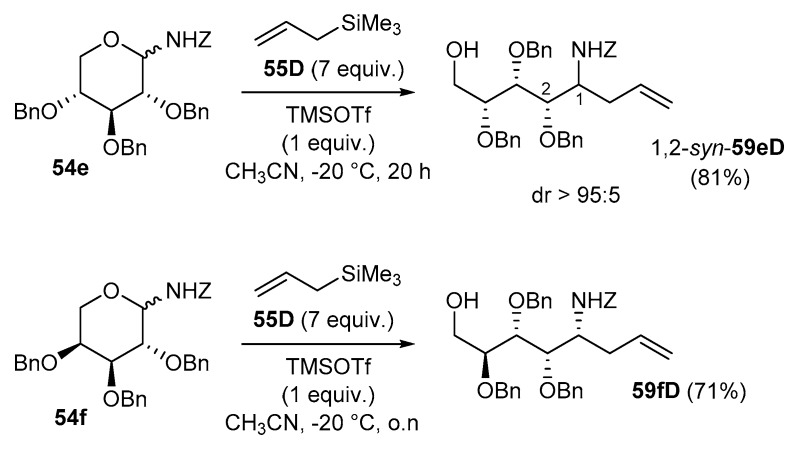
Ring-opening reactions of *N*-Z-pentopyranosylamines **54e** and **54f** [92].

As expected, addition to *N*-Z-pentopyranosylamines proceeds more efficiently to give related alditols in good yields and good diastereoselectivities (see **59eD** and **59fD**, Scheme 22) [92].

### 4.3. Cyclization Reactions

The open-chain silylated iminoalditols were generally cyclized via a mesylation/*t*-BuOK-treatment sequence. The cyclization step requires a stronger base than in the case of the *N*-alkylated aminoalditols. Cyclization occurs also through an intramolecular S_N_2 reaction with inversion at C-4 or C-5. The related protected 1,2-*cis* iminosugar-*C*-glycosides are usually obtained in good yields. Of note, retention of the configuration at the carbon atom carrying the free OH group could also be achieved by a sequence of oxidation—intramolecular reductive amination.

**Figure 3:**
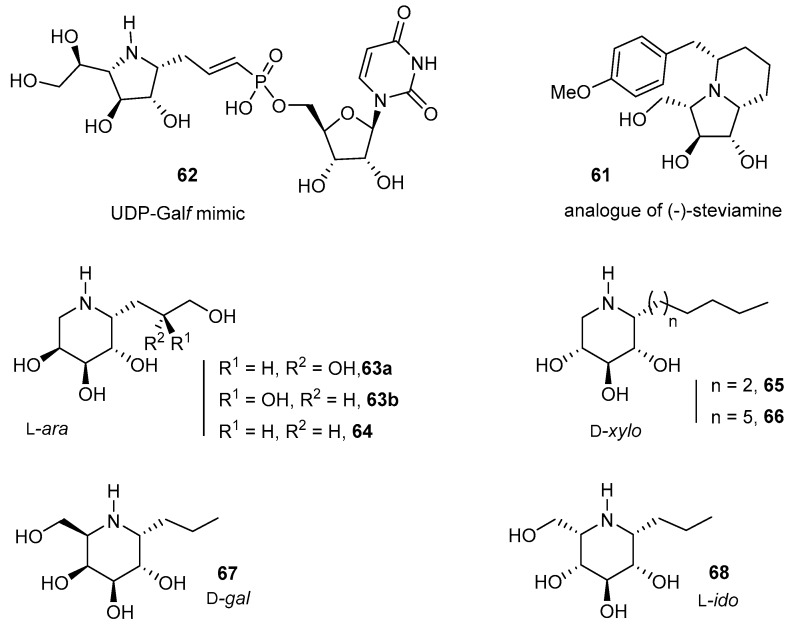
Iminosugar-*C*-glycosyl compounds.

These valuable reactions have been applied to a range of furanosides and a few pyranosides to give a new poly-hydroxylated indolizidine derivative **61** as an analogue of (–)-steviamine [95], UDP-Gal*f* mimics such as **62** [90,91], or potent pharmalogical chaperones (in the l-*arabino*, d-*xylo*, d-*galacto* and l-*ido* iminosugar-*C*-glycoside series, see **63**–**68**) for the treatment of glucosidase and galactosidase-linked lysosomal storage disorders (LSDs) (Figure 3) [92,94].

### 4.4. Miscellaneous

As miscellaneous examples, 2-deoxy glycosylamines of type **69** could be synthesized through vicinal amino chlorination of related glycals **70** [96].

**Scheme 23:**
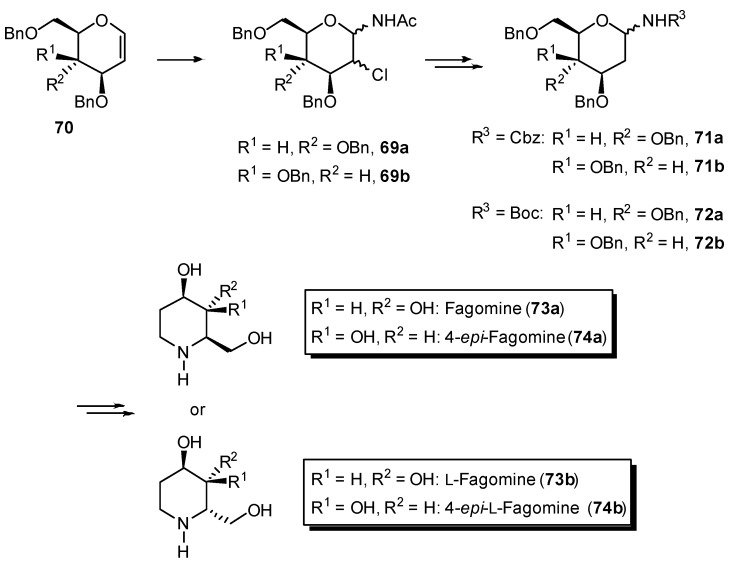
Synthesis of fagomines and their analogues [96,97].

Following a dechlorination protocol, unmasking of the amide functionality and protection of the free amine as its benzyl- or *tert*-butyl carbamate, glycosylamines **71** and **72** were prepared. Reduction of the *N*-oxycarbonyl-*N*-glycosides with LiAlH_4_ followed by cyclization-deprotection gave fagomines **73a**–**b** and their epimers **74a**–**b** (Scheme 23) [97].

**Scheme 24:**
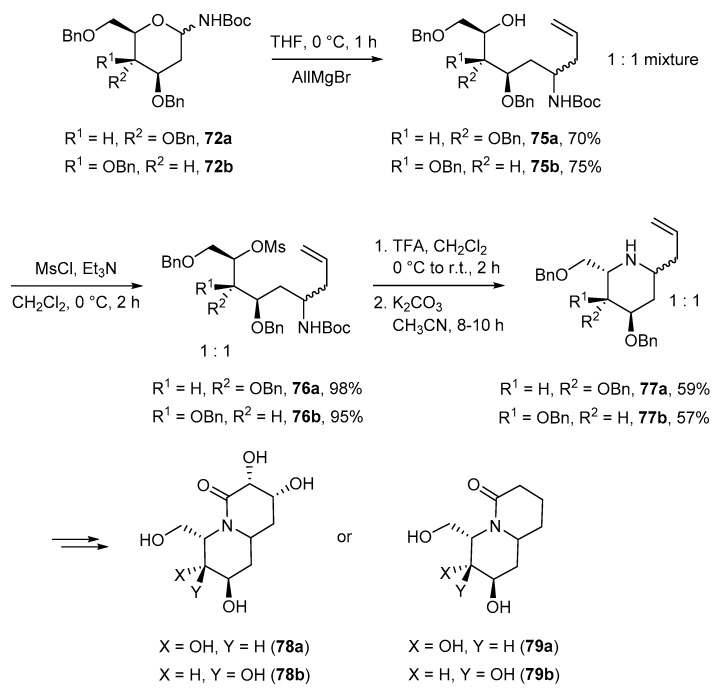
Synthesis of novel polyhydroxylated quinolizidines **78** and **79**.

Interestingly, *N*-Boc-protected glycosylamines **72a** and **72b**, obtained, respectively, from d-glucal and d-galactal derivatives **70a** and **70b** could also be treated with excess allylmagnesium bromide to give the ring-opened amino alcohols **75a** and **75b** in good yields (70% and 75%, respectively) as an inseparable 1:1 mixture of diastereomers (Scheme 24) [98]. The free hydroxyl groups of amino alcohols **75a** and **75b** were mesylated and cyclized under intramolecular S_N_2 reactions (after removal of the Boc group and treatment with K_2_CO_3_) to give compounds **76** and **77**, respectively. After separation of the diastereomers and further elaboration, novel polyhydroxylated quinolizidines of type **78** and **79** were provided in good yields. These molecules could be regarded as analogues of l-1,2-dideoxyhomonojirimycin.

## 5. *N*-(*tert*-Butanesulfinyl) Glycosylamines

Considering the higher stability of the *N*-*tert*-butanesulfinyl aldimines and ketimines compared with most *N*-alkyl, aryl, acyl, and carbonyl Schiff’s bases, as well as the advantages of Ellman’s imines in terms of stereocontrol, *N*-*tert*-butanesulfinyl glycosylamines have recently emerged as more versatile synthetic intermediates en route to imino-*C*-glycosyl compounds.

### 5.1. Synthesis

**Scheme 25:**
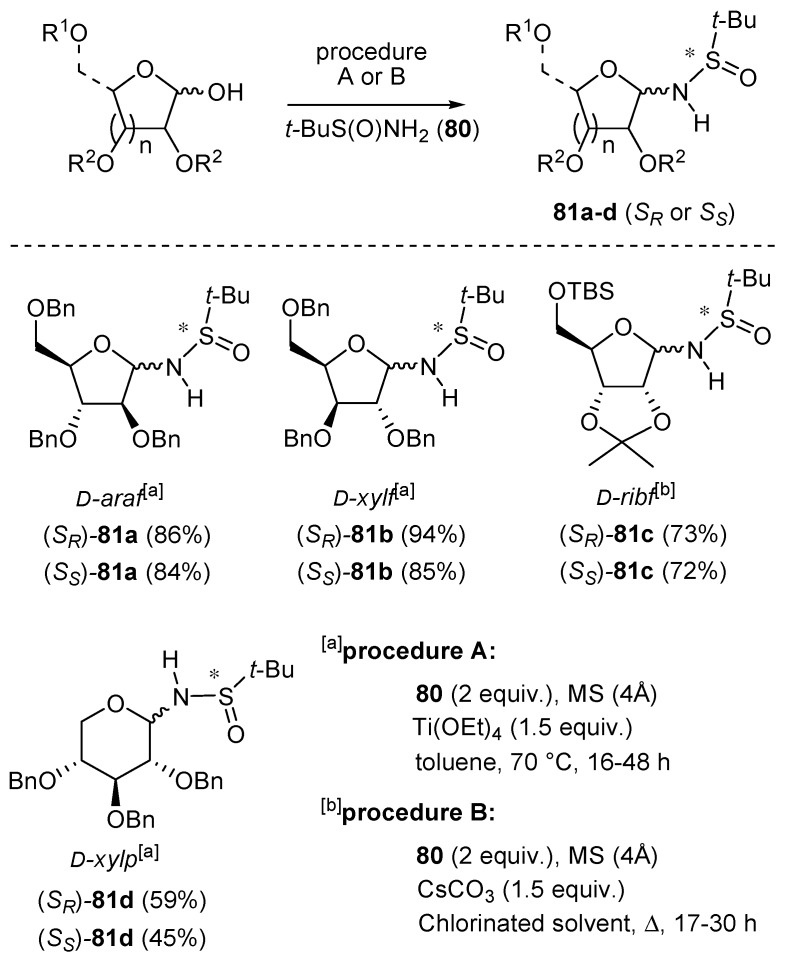
Preparation of sulfinylglycosylamines **81a**–**81d** [99,100,101].

As shown in Scheme 25 (see procedure A), these derivatives are commonly synthesized by the addition of an *O*-benzyl-protected pentofuranose or pentopyranose to a mixture of (*R*)- or (*S*)-2-methyl-2-propanesulfinamide **80** (2 equiv.) and Ti(OEt)_4_ (1.5 equiv.) in dry toluene. The reaction mixture is heated at 70 °C to give compounds **81a**, **81b** and **81d** in moderate to excellent yields (45–94%) [99,100,101].

Alternatively, *N*-sulfinyl glycosylamines with different protecting groups may be prepared using a modified protocol (see procedure B). For example, *N*-*tert*-butanesulfinyl ribofuranosyl-amines (*S_R_*)-**81c** and (*S_S_*)-**81c**, carrying acid-sensitive groups were prepared in moderate yields by reacting the substrate with (*R*)- or (*S*)-Ellman’s sulfinamide in the presence of CsCO_3_ and molecular sieves for 17–30 h under reflux [99,100].

Interestingly, the *N*-sulfinylglycosylamines are hydrolytically stable (particularly, in the furanose form), allowing their isolation by normal silica gel (SiO_2_) chromatography. Furthermore, the two anomers (α/β) of compounds **81** could be separated (SiO_2_ chromatography), although a slow epimerization was observed when the anomers of (S*_R_*)-**81a** were separately analyzed by NMR spectroscopy as solutions in CDCl_3_. These glycosylamines can be handled in air at room temperature, but prolonged storage at room temperature results in decomposition over a period of a few days. Stability is greatly increased by storing the *N*-sulfinyl glycosylamines in closed containers at 5 °C [99].

### 5.2. Addition Reactions

The *N*-*tert*-butanesulfinyl glycosylamines undergo addition reactions with a range of Grignard and lithium reagents to give related 1,2-*syn* or 1,2-*anti*-aminoalditols in good yields and moderate to excellent diastereoselectivities [99,101,102,103]. Their reactivity was found to be similar to that of *N*-alkyl-*N*-glycosides and of *N*-benzyl-*N*-glycosylhydroxylamines with a chemical stability similar to that of the *N*-Cbz-analogues [99]. In that respect, and as shown by the potential of the chiral sulfinyl auxiliary to direct in some instances the stereoselectivity at C-1, they provide more reliable and versatile synthetic intermediate en route to iminosugar-*C*-glycosides than previously described *N*-alkyl and *N*-alkoxycarbonyl glycosylamines.

### 5.3. Addition of Grignard Reagents and Cyclizations

As reported in Table 3, the addition of Grignard reagents (e.g., alk, Ar, all, Bn, propargyl, vinyl, etc.) proceeded usually by adding an excess of the organometallic species to a solution of a *N*-*tert*-butanesulfinyl-*N*-glycoside in THF at −60 °C. The reaction mixture is subsequently allowed to reach −20 °C, over 1.5 h to give the related 1,2-*syn* aminoalditol derivative **82** in good yield (45–95%) and moderate to excellent diastereoselectivity.

**Scheme 26:**
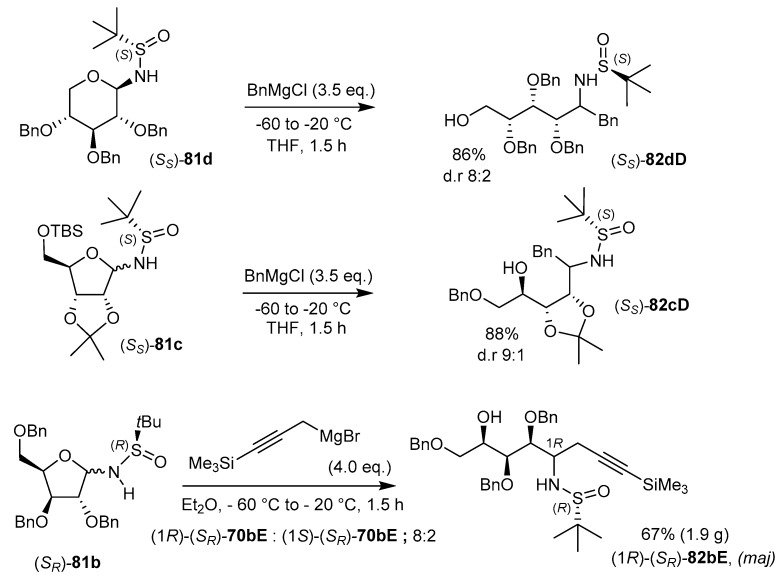
Addition of Grignard reagents onto *N*-*tert*-butanesulfinyl glycosylamines [99,101].

Interestingly, the diastereomeric ratio is often comprised between 7:3 and 10:0 in favor of the 1,2-*syn* diastereomer, from either epimer at the S-atom ((*S_S_*) or (*S_R_*)). Thus, in such cases, the chiral *N*-*tert*-butanesulfinyl auxiliary does not direct the stereoselectivity at C-1. Importantly, however, the process can be scaled-up (up to 1.9 g of product **82**) [102], without erosion of the diastereo-selectivity. The method was implemented to a variety of organomagnesium species (see Table 3) [99], including propargyl Grignard reagents [101]. It may be possible to further enrich the diastereoselectivity significantly by adding LiCl, which resulted in d.r. greater than 90% (entries 3, 7, 12). The addition reactions were extended to different series, namely on glycosylamines derived from 2,3,4-tri-*O*-benzyl-d-xylopyranose [99] (e.g., **81d**), 2,3,5-tri-*O*-benzyl-d-xylofuranose (e.g., **81b**), [101], and the acid-sensitive furanose derivative **81c** [99] to afford compounds (*S_S_*)-**82dD**, (*S_R_*)-**82bE**, and (*S_S_*)-**82cD**, respectively, in good yields and good diastereoselectivities (Scheme 26).

Furthermore, although in these series the chiral sulfinyl group does not control the stereochemistry, the diastereoisomers of the 1-*C*-substituted iminoalditols were all separable by regular SiO_2_-column chromatography using either (*S_S_*)-*N*- or (*S_R_*)-*N*- or both sulfinyl glycosylamines. This is remarkable since, in the *N*-benzyl- and other *N*-alkyl-*N*-glycoside series, as well as in *N*-benzylhydroxylamines and *N*-alkoxycarbonylglycosylamines, difficulties in separating both diastereomers often hampered the synthetic utility of these important scaffolds.

**Scheme 27:**
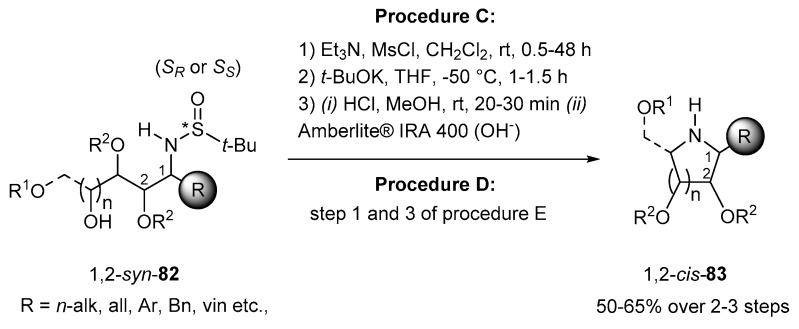
Synthesis of iminosugar-*C*-glycosides from *N*-*t*-butanesulfinyl iminoalditols [99,101,102,103].

As illustrated in Scheme 27, the *N*-*tert*-butanesulfinyl iminoalditols were routinely cyclized under the same conditions as those reported for *N*-Cbz-*N*-glycosides (mesylation followed by treatment with *t*-BuOK). Afterwards, the sulfinyl protecting group was removed with mild acid (HCl in MeOH) to generate the corresponding 1,2-*cis* imino-*C*-glycosyl derivatives **83** in good yields (see procedure C) [99,102]. Alternatively, the sulfinyl group may be cleaved first and cyclization promoted from the free amine, which occurs spontaneously (procedure D) [101,103].

**Scheme 28:**
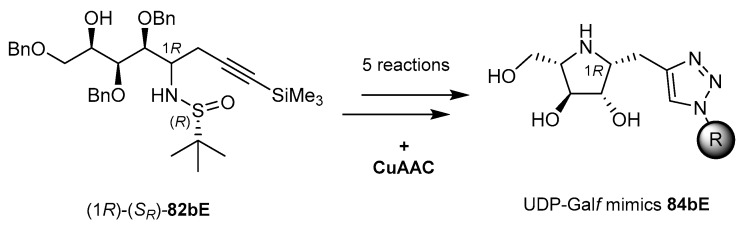
Synthesis of triazole-linked iminosugars and aromatic systems as simplified UDP-Galf mimics [102].

The preparation of 1,4-dideoxy-1,4-imino-l-arabinitol scaffolds **84bE** tethered to 1,2,3-triazoles carrying (hetero)aromatic systems as simplified uridinyl diphospho-d-galactofuranose (UDP-Gal*f*) mimics were prepared by these methods through the addition of a trimethylsilylpropargyl Grignard reagent to *N*-sulfinylglycosylamine (*S_R_*)-**81b**, followed by cyclization/deprotection sequences (Scheme 28) [102]. Compound **84bE** is a moderate inhibitor of *GlFT2*, a key galactofuranosyltransferase involved in the assembly of the cell wall of mycobacteria (including the causative agent of tuberculosis, *Mycobacterium tuberculosis*) [104], and it is essential for mycobacterial viability [105,106].

### 5.4. Addition of M-CF_2_P(O)(OEt)_2_ Metalated Species and Cyclizations

**Scheme 29:**
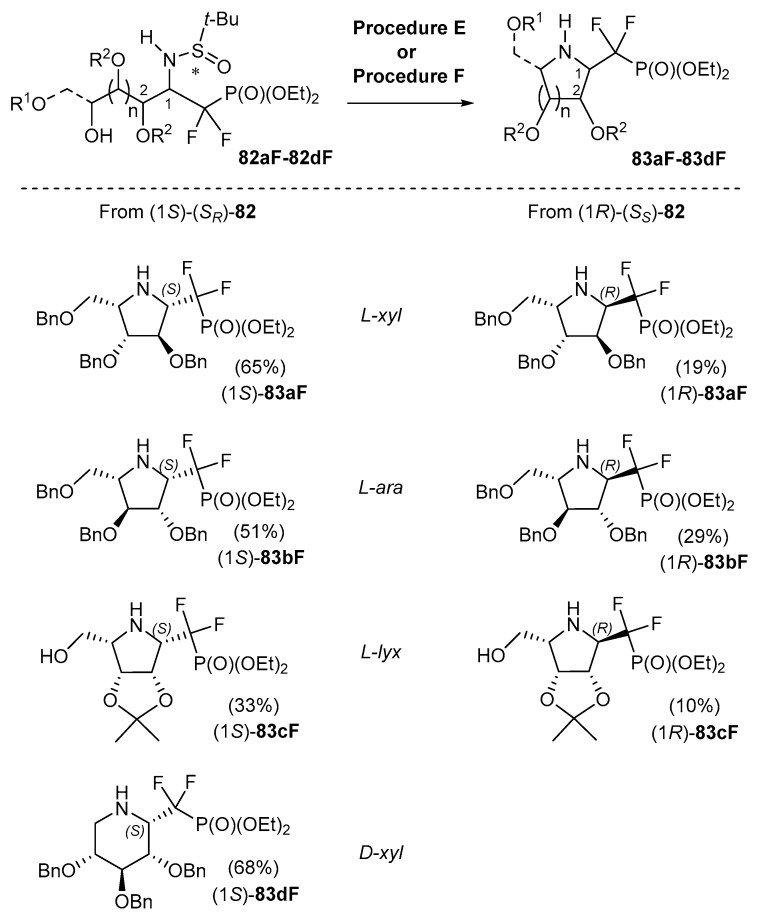
Tunable synthesis of 1-*C*-diethylphosphono(difluoromethyl) iminosugars as glycosyl phosphate mimics [101].

We have recently described an efficient methodology for the introduction of a CF_2_P(O)(OEt)_2_ group by the addition of either BrMgCF_2_P(O)(OEt)_2_ (prepared by reacting BrCF_2_P(O)(OEt)_2_ with *i*-PrMgCl and LiBr in THF at −75 °C = procedure E) or LiCF_2_P(O)(OEt)_2_ (generated from LDA and HCF_2_P(O)(OEt)_2_ in THF at −60 °C = procedure F) [101] (Scheme 29).

Upon activation/cyclization, the resulting aminoalditols **82aF**–**82dF** lead to 1-*C*-difluorophosphonomethyl-iminosugar-*C*-glycosides **83aF**–**83dF** in modest to good overall yields (ca. 3–49% over 3–4 steps). Such compounds are very important mimics of glycosyl phosphates and precursors of sugar nucleotide analogs. Remarkably, the stereoselectivity of the addition of these reagents is tunable, i.e., the pseudoanomeric configuration of the glycosyl phosphate mimics can be chosen by selecting the configuration of the sulfinyl group in the starting *N*-*t*-butanesulfinyl glycosylamines. The corresponding *N*-*t*-butanesulfinyl iminoalditol derivatives **82aF**–**82dF** were obtained in moderate to good yields (44–88%) and modest to excellent diastereoselectivities (6:4 to 10:0) from compounds **81a**–**d**. Details on the stereochemical effects at play in this process were gained from quantum chemical calculations [101]. These can be exploited to predict the selectivities of future novel substrates. As a rule, glycosylamines (*S_S_*)-**81** give (1*R*)-(*S_S_*)-**83aF**–**dF** (i.e., a pseudo α-anomer) and (*S_R_*)-**81**, (1*S*)-(*S_R_*)-**83aF**–**dF** (i.e., a pseudo β-anomer) respectively, as the major products.

**Scheme 30:**
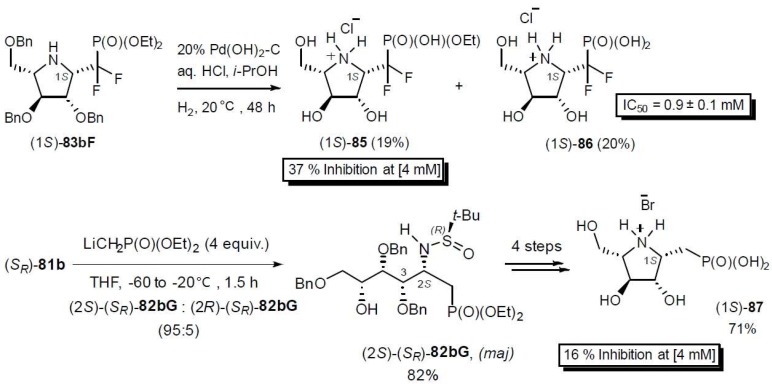
Synthesis of 1-*C*-Difluorophosphonomethyl- and 1-*C*-phosphonomethyl-1,4-imino-l-arabinitols and their activity as Gal*f* transferase inhibitors [101,103].

Compound (1*S*)-**83bF** was deprotected by hydrogenation using Pd(OH)_2_/C (20%) as the catalyst [103]. Introduction of the −CH_2_P(O)(OEt)_2_ moiety was also performed using similar synthetic sequences from (*S_R_*)-**81b** and LiCH_2_P(O)(OEt)_2_ [103]. β-Phosphonomethyl- and β-phosphono(difluoromethyl)-1,4-imino-l-arabinitols (1*S*)-**85**, (1*S*)-**86**, and (1*S*)-**87** were provided in low to moderate overall yields. Compounds **85**–**87** were found to be moderate inhibitors of the mycobacterial galactofuranosyltransferase Gal*f*T2 (Scheme 30).

## 6. Conclusions

Since the pioneering studies of Nicotra and co-workers in the early 1990s, and more recent contributions from Kobayashi and Dondoni, *N*-protected glycosylamines have progressively made their way to become important synthetic scaffolds en route to iminosugar-*C*-glycosyl compounds. Various types of *N*-glycosyl derivatives (e.g., in the pentofuranose, pentopyranose, and hexopyranose series) have been reported in the last 20 years and the synthetic reaction sequences giving important iminosugar-*C*-glycosyl derivatives have frequently been improved. Foremost, they include the direct condensation of a primary amine with a protected sugar hemiacetal, followed by a typical addition, activation, and cyclization reaction sequence. In particular, the valuable *N*-*tert*-butanesulfinyl glycosylamines have very recently been developed giving an approach to iminosugar-*C*-glycosides where the stereoselectivity at C-1 may be tuned. In that respect, this field of research will, hence, surely continue to motivate the scientific community in designing new types of *N*-glycosidic structures for the synthesis of iminosugars, as well as for their use for therapeutic purposes.

## Figures and Tables

**Table 1:** Addition of organometallic reagents R-[M] to *N*-Benzyl-*N*-glycosyl-hydroxylamines [77,78,80,81].

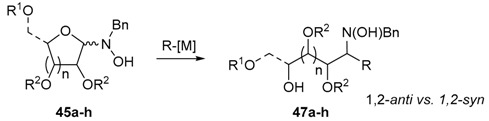				
Entry	Hydroxylamine	R-[M]	Yield (%) ^[a]^	*anti*/*syn* ^[b]^
1	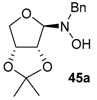	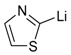	65	98/2
2	**45a**	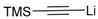	72	70/30
3	**45a**	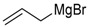	95	96/4
4	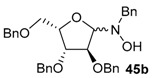	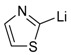	72	80/20
5	**45b**	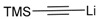	82	90/10
6	**45b**	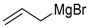	80	85/15
7	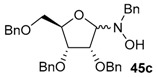	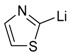	72 ^[c]^	ND ^[d]^
8	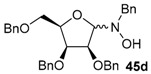	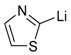	52 ^[c]^	70/30
9	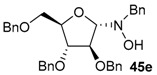	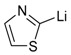	75	90/10
10	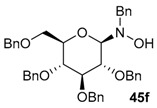	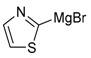	80	25/75
11	**45f**	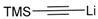	85	85/15
12	**45f**	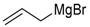	85	50/50
13	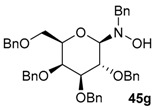	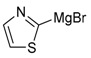	77	33/67
14	**45g**	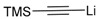	85	75/25
15	**45g**	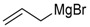	90	50/50
16	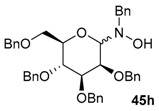	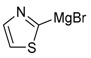	82	60/40
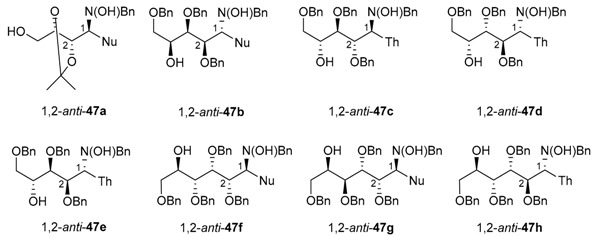				

^[a]^ Isolated yield of the diastereomeric mixture after flash chromatography; ^[b]^ The diastereomeric ratio was determined by ^1^H-NMR analysis of the crude mixture; ^[c]^ Yield of isolated 1,2-*anti* disatereomer; ^[d]^ ND = not determined.

**Table 2:** Addition of silicon reagents **55A**–**D** (Nu-SiR_3_) to *N*-(benzyloxycarbonyl)-*N*-glycosides **54a**–**d** [89].

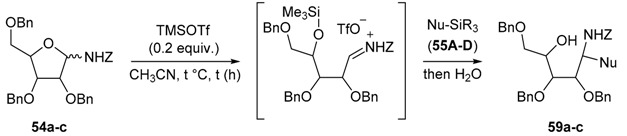			
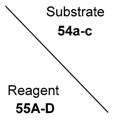	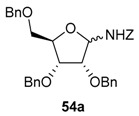	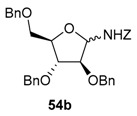	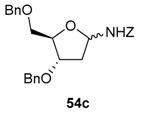
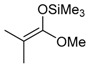 **55A**	−40 °C, 12 h ^[a]^ **59aA** (quant.) ^[b,d]^ *syn*:*anti* 93:7	−40 °C, 21 h ^[a]^ **59bA** (62%) ^[b,d]^ *syn*:*anti* 91:9	-
Me_2_ClSiH **55B**	0 °C, 6 h ^[c]^ **59aB** (76%) ^[b,d]^	0 °C, 9 h ^[c]^ **59bB** (59%) ^[b,d]^	-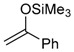 **55C**	−40 °C, 16 h ^[a]^ **59aC** (70%) ^[b,d]^ *syn*:*anti* 93:7	−40 °C, 37 h ^[a]^ **59bC** (77%) ^[b,d]^ *syn*:*anti* 93:7	−40 °C, 9 h ^[a]^ **59cC** (80%) ^[b,d]^ *syn*:*anti* 50:50
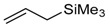 **55D**	0 °C, 23 h ^[a]^ **59aD** (0%) ^[b,d]^	−30 °C, 39 h ^[a]^ **59bD** (64%) ^[b,d]^ *syn*:*anti* > 94:6	-
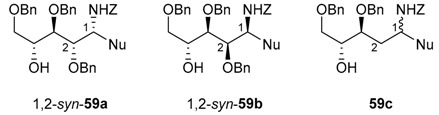			

^[a]^ Reactions were performed using 0.2 equiv. of TMSOTf and 2 equiv. of silylated nucleophile; ^[b]^ Isolated yield of the diastereomeric mixture after flash chromatography; ^[c]^ Reactions were performed using 1.0 equiv. of TMSOTf and 2 equiv. of Me_2_ClSiH; ^[d]^ Product **59**: **A** Nu = CMe_2_CO_2_Me; **B** Nu = H; **C** Nu = CH_2_COPh; **D** Nu = CH_2_CH = CH_2_.

**Table 3:** Addition of Grignard reagents onto *N*-*tert*-butanesulfinyl glycosylamines (*S_R_*)-**81a** and (*S_S_*)-**81a** [99].

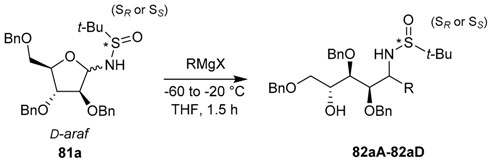					
Entry	Reagent (equiv.)	Auxiliary (S*_R_* or S*_S_*)	Dr ^[a]^	Yield (%) ^[b,c]^	R*f* ^[d]^
1	PhMgBr (3.5)	*S_R_*	7:3	69	0.3
2	PhMgBr (3.5)	*S_R_*	10:0 ^[e]^	30	–
3	PhMgBr (3.5)	*S_R_*	85:15 ^[e,f]^	60	–
4	vinMgBr (6)	*S_R_*	8:2	69	0.5/0.3
5	*n*-hexMgBr (6)	*S_R_*	6:4	45	0.5
6	BnMgCl (3.5)	*S_R_*	7:3	83	0.6/0.55
7	BnMgCl (3.5)	*S_R_*	9:1 ^[e]^	95	
8	PhMgBr (3.5)	*S_S_*	7:3	57	0.5/0.2
9	vinMgBr (6)	*S_S_*	8:2	62	0.3/0.1
10	*n-*hexMgBr (6)	*S_S_*	6:4	40	0.5/0.4
11	BnMgCl (3.5)	*S_S_*	~85:15	94	0.6/0.5
12	BnMgCl (3.5)	*S_S_*	97:3 ^[e]^	83	–
13	allMgBr (3.5)	*S_S_*	5:5	83	0.6/0.4

^[a]^ d.r determined on crude mixture using ^1^H-NMR (250 MHz) spectroscopy; ^[b]^ Isolated yield (SiO_2_ column chromatography); ^[c^^]^ **82aA** (R = Ph), **82aB** (R = vin), **82aC** (R = *n*-hex), **82aD** (R = Bn); ^[d]^ (1st eluted fraction/2nd eluted fraction). R*f* determined on TLC (SiO_2_) using PE:EA 5:5 as eluent; ^[e]^ Reaction performed with LiCl (3.5 equiv.) as an additive; ^[f]^ Reaction mixture allowed to reach 5 °C over a period of 3.5 h.
